# Aberrant Right Subclavian Artery-Induced Esophageal Stenosis Successfully Treated with Vascular Reconstruction: A Pediatric Case Report

**DOI:** 10.70352/scrj.cr.25-0182

**Published:** 2025-09-25

**Authors:** Wataru Sumida, Kazuya Nagayabu, Kazuki Ota, Yuki Imai, Yasuyuki Ono, Hiroomi Murayama

**Affiliations:** 1 Department of Pediatric Surgery, Aichi Children’s Health and Medical Center, Obu, Aichi, Japan; 2 Department of Pediatric Cardiology, Aichi Children’s Health and Medical Center, Obu, Aichi, Japan; 3 Department of Pediatric Cardiovascular Surgery, Aichi Children’s Health and Medical Center, Obu, Aichi, Japan

**Keywords:** aberrant right subclavian artery, acute respiratory failure, tracheomalacia, esophageal foreign body

## Abstract

**INTRODUCTION:**

Aberrant right subclavian artery (ARSA) is the most common congenital anomaly of the subclavian artery, occurring in 0.2%–1.5% of the population. While typically asymptomatic, ARSA can cause dysphagia in older patients due to esophageal compression. In pediatric cases, it is less frequent and usually presents with chronic respiratory symptoms resulting from tracheal compression. Acute respiratory failure as a presentation of ARSA is extremely rare. This case report describes a pediatric patient with ARSA-associated esophageal stricture, leading to severe respiratory distress following food intake and requiring surgical treatment.

**CASE PRESENTATION:**

A 1.8-year-old girl developed an inspiratory stridor after eating. Initial inhalation therapy provided temporary relief, but persistent respiratory distress prompted further evaluation. CT revealed an esophageal foreign body, which was successfully extracted using a balloon catheter. However, continued respiratory distress necessitated intubation and intensive care. Bronchoscopy and enhanced CT confirmed tracheal compression due to ARSA, along with esophageal stenosis. Despite initial medical management, recurrent respiratory distress and feeding difficulties required surgical intervention. A right thoracotomy was performed to reposition the aberrant artery anterior to the trachea, alleviating the vascular compression. Postoperatively, the patient made a full recovery, resumed a normal diet, and remained symptom-free at the 6-month follow-up.

**CONCLUSIONS:**

Symptomatic ARSA is rare in pediatric patients but can cause severe respiratory distress due to esophageal and tracheal compression. In cases of recurrent or severe symptoms, surgical intervention is essential to prevent life-threatening complications. This case highlights the importance of considering ARSA in pediatric patients with unexplained respiratory distress, especially when associated with feeding difficulties.

## Abbreviations

ARSA
: aberrant right subclavian artery
SD
: standard deviation

## INTRODUCTION

Variations in the anatomy of the aortic arch are common in the general population and are often detected incidentally. A left-sided aortic arch with an ARSA is the most frequently occurring congenital anomaly of the subclavian artery, affecting 0.2%–1.5% of the population.^1)^

Most ARSA cases remain asymptomatic and are identified incidentally. When symptoms do occur, they typically involve dysphagia resulting from esophageal compression by an abnormally positioned blood vessel. Dysphagia is more commonly observed in older patients, as arteriosclerosis can exacerbate the compression-related symptoms.^2)^ By contrast, ARSA is seldom diagnosed in pediatric patients. However, when symptoms manifest, they have been documented to primarily manifest as chronic airway symptoms. The etiology of respiratory symptoms in children is believed to be attributed to both the abnormal vascular course and the relative lack of tracheal rigidity during early childhood.^3)^

This report presents a pediatric case of acute respiratory failure triggered by an esophageal foreign body. The foreign body resulted from esophageal stricture caused by posterior compression of the esophagus by ARSA, ultimate necessitating surgical intervention.

## CASE PRESENTATION

A 1.8-year-old girl with no known medical history was transferred to our hospital due to inspiratory stridor following food intake. At admission, her body weight and height were 9.5 kg (+0.2 SD) and 81 cm (+1.7 SD), respectively. This finding indicated an absence of growth retardation, thereby suggesting that the patient did not have a chronic eating disorder.

The stridor first appeared after she consumed a meal, one before her transfer. She initially visited the emergency department at a local hospital, where inhalation therapy provided temporary symptom relief. However, as the stridor persisted, she sought further evaluation at a pediatric outpatient clinic the next day. A CT scan revealed an esophageal foreign body, prompting her transfer to our hospital for additional evaluation and treatment.

**Fig. 1:**
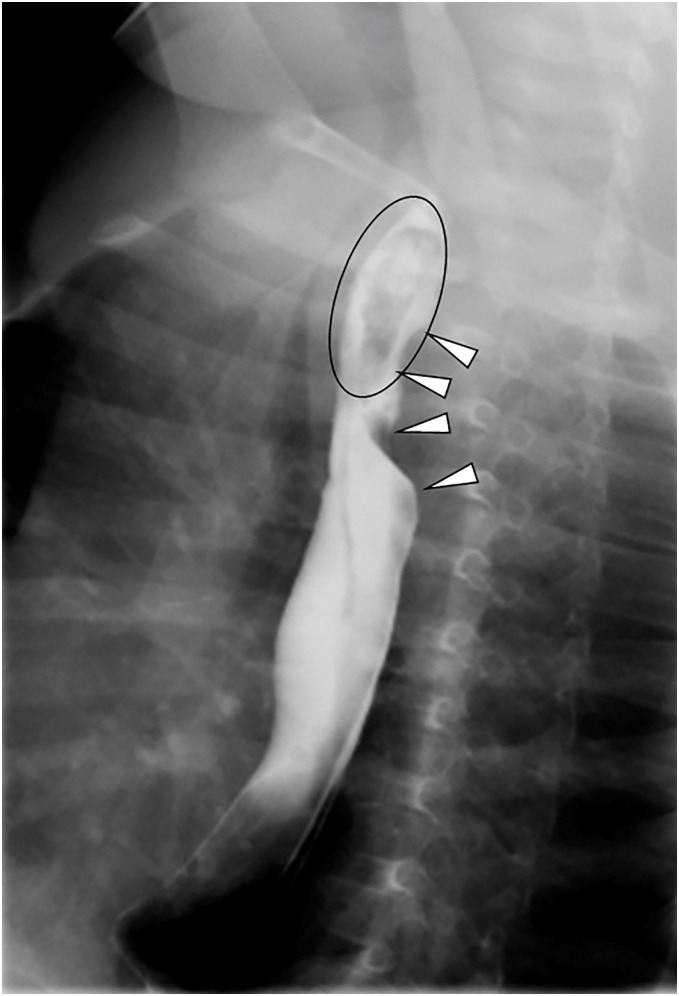
Fluoroscopic imaging at the initial emergency room consultation. The circle highlights the foreign body (a piece of eggplant), while the white arrowheads indicate esophageal compression from the dorsal side.

Upon arrival, the patient exhibited significant respiratory distress, characterized by accessory muscle use, retractions, and pronounced inspiratory stridor. Given the previous hospital’s CT findings indicating an esophageal foreign body, an emergency fluoroscopic examination was conducted (**Fig. 1**). The foreign body, a piece of eggplant, was successfully extracted using a balloon catheter. Additionally, the esophagus showed evidence of external compression from the dorsal side.

**Fig. 2:**
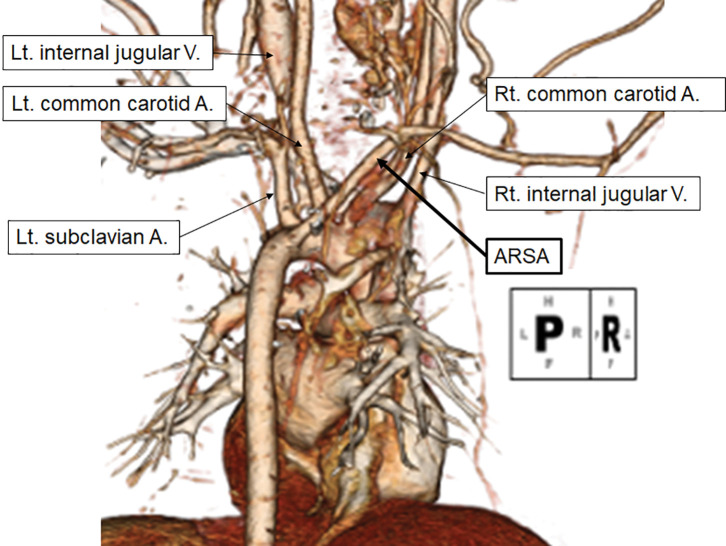
Dorsal view of the CT angiogram from the 1st hospital admission. The 1st branch of the aortic arch formed only the common carotid artery, while the 4th branch gave rise to the ARSA. A, artery; ARSA, aberrant right subclavian artery; Lt, left; Rt, right; V, vein

Despite the immediate relief of the esophageal obstruction, the patient’s respiratory distress persisted, necessitating endotracheal intubation and close monitoring in the pediatric ICU. To determine the underlying cause, she underwent flexible bronchoscopy and dynamic enhanced CT imaging. Bronchoscopy revealed external tracheal compression along with tracheomalacia. Enhanced CT imaging demonstrated that the 1st branch of the aortic arch gave rise only to the common carotid artery, while the 4th branch coursed posterior to the esophagus before forming the right subclavian artery (**Fig. 2**), consistent with ARSA.

After a 5-day course of intravenous steroids and inhaled epinephrine, the patient’s respiratory symptoms temporarily stabilized. She was successfully extubated. Given the improvement of her symptoms following the medical therapy, the decision was made to discharge her without undergoing surgical intervention, as the symptoms were deemed to be transient. She was subsequently discharged from the hospital.

Approximately 1 month after discharge, she developed worsening symptoms of poor oral intake and dyspnea. Eventually, she presented to our hospital’s emergency department with respiratory distress triggered by solid food intake.

**Fig. 3:**
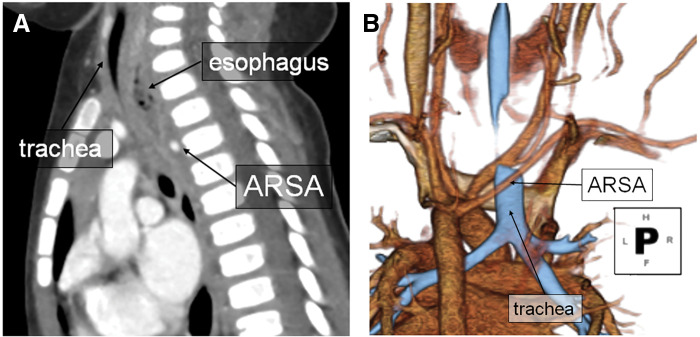
Images of CT scan on the 2nd hospital admission. The image reveals esophageal stenosis due to ARSA compression, along with esophageal dilatation proximal to the compression site. Additionally, the trachea appears narrowed due to compression from the dilated esophagus. (**A**) Sagittal view (**B**) 3D reconstruction image. ARSA, aberrant right subclavian artery

A CT scan revealed esophageal stenosis, characterized by wall thickening and dilation on the oral side due to compression from ARSA. Additionally, the trachea was compressed by the dilated and thickened esophagus, resulting in tracheal stenosis (**Fig. 3**). Based on these findings, we concluded that the severe stridor in the present case was due to narrowing of the trachea caused by compression of the trachea by the dilated esophagus. The contents found in the esophagus were consistent with masticated food, rather than a solid state. It was determined that the condition had progressed since the previous episode. Surgical intervention was deemed necessary for definitive resolution of the respiratory condition.

**Fig. 4:**
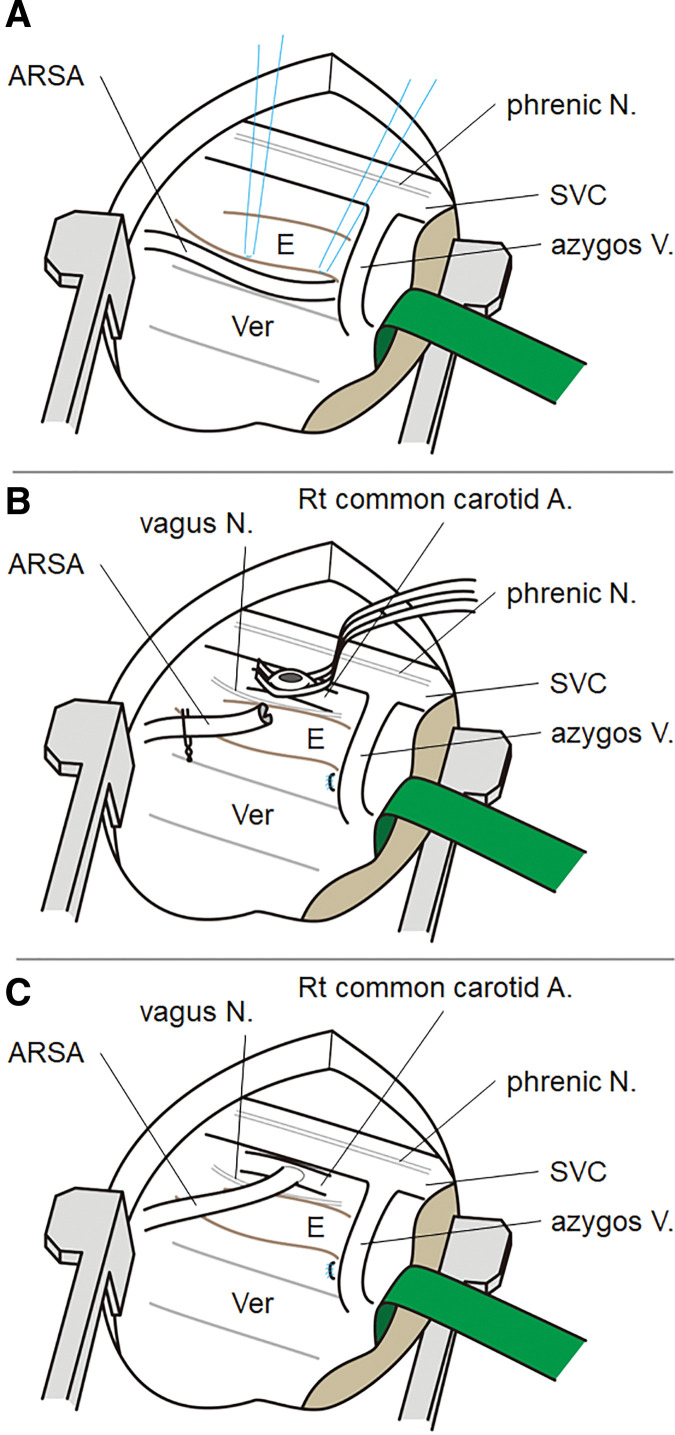
Schematic representation of the surgical procedure. (**A**) ARSA was identified posterior to the esophagus following a right thoracotomy. The 4 blue lines indicated threads for traction. (**B**) ARSA was transected, its central end was closed using double running sutures with 6-0 Prolene, and the right common carotid artery was side-clamped. A longitudinal incision was made at the clamped site. (**C**) ARSA was rerouted and anastomosed to the right common carotid artery in an end-to-side fashion. A, artery; ARSA, aberrant right subclavian artery; E, esophagus; N, nerve; Prolene, Johnson & Johnson, New Brunswick, NJ, USA; SVC, supra vena cava; V, vein; Ver, vertebra

A right thoracotomy was performed at the 3rd intercostal space to access the ARSA, which was identified as originating from the descending aorta. The ARSA was observed to pass posterior to the esophagus. ARSA was transected at the point of sufficient length for reconstruction, and its central portion was closed using double 6-0 Prolene sutures (Johnson & Johnson, New Brunswick, NJ, USA). The aberrant artery was then repositioned anterior to the trachea. The right common carotid artery was temporarily side-clamped while monitoring cerebral oxygen saturation to ensure stability during the procedure. A longitudinal incision was made at the clamped site, and the ARSA was anastomosed to the right common carotid artery in an end-to-side configuration using 7-0 Prolene sutures (**Fig. 4**).

The postoperative course was uneventful. Fluoroscopic evaluation confirmed adequate esophageal clearance, allowing the patient to resume a normal diet.

**Fig. 5:**
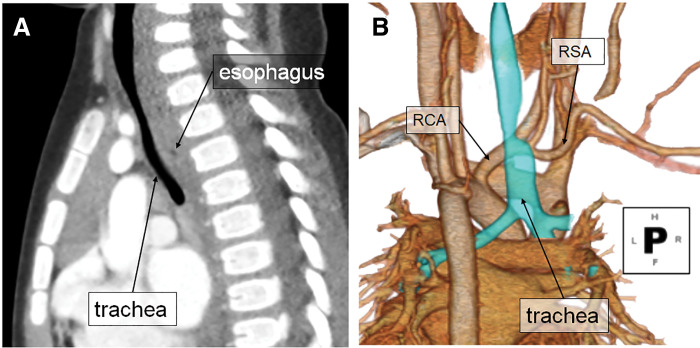
Images of CT scan examined 6 months after surgical intervention. ARSA was not detected. The RSA is anastomosed to the RCA and passes anterior to the trachea. The esophageal dilation had been reduced, and tracheal stenosis had been reduced. (**A**) Sagittal view (**B**) 3D reconstruction image. ARSA, aberrant right subclavian artery; RCA, right common carotid artery; RSA, right subclavian artery

At the 6-month follow-up, a CT scan was performed to assess the surgical outcome (**Fig. 5**). The scan showed no stenosis at the reconstructed vessel site and no airway deformity. The patient remained asymptomatic, with no respiratory difficulties or oral intake restrictions in daily life.

## DISCUSSION

ARSA was first described by Hunald in 1735, and in 1787, Bayford identified its association with dysphagia, later termed dysphagia lusoria.^4)^ ARSA is the most common congenital anomaly of the aortic arch, with a reported prevalence of 0.2%–1.5% in the general population, according to a systemic review.^1)^ In individuals with ARSA, 4 major vessels arise from the aortic arch, with the aberrant artery originating as the 4th branch from the thoracic aorta, distal to the left subclavian artery. ARSA typically follows an abnormal course, passing posterior to the esophagus in approximately 80% of cases. Less commonly, it traverses between the trachea and the esophagus (13%) or follows a peritracheal route (5%).^5)^

Most ARSA cases remain asymptomatic, with over half of affected individuals never developing symptoms. The anomaly is often detected incidentally through imaging studies or autopsy.^5)^ However, when symptoms do occur, more than 70% of patients experience dysphagia due to esophageal compression caused by the aberrant artery. Dysphagia is more frequently observed in older patients, as atherosclerosis can exacerbate the compression.^2)^

By contrast, ARSA cases in children are uncommon and are primarily associated with chronic respiratory symptoms. The higher prevalence of respiratory issues in pediatric patients has been linked to the reduced tracheal rigidity in early childhood compared with adults.^3)^

A literature review of 25 pediatric ARSA cases was conducted in 2012.^6)^ Upon analysis, it was reported that 14 of the 25 cases presented with respiratory symptoms. Further analysis of the literature indicated that 9 of the 14 cases with respiratory symptoms were attributed to infection. Furthermore, in 6 of those 9 cases, the esophagus was revealed to be dilated or compressed posteriorly. Although not described in detail in the literature, it was assumed that these cases could have caused aspiration due to impaired esophageal transit. To further investigate symptomatic pediatric ARSA cases, a literature review was conducted on studies published after the 2012 review article. A PubMed search identified 18 cases across 13 reports published since 2013.^7–19)^ The findings indicated that dysphagia was the most commonly reported symptom, while respiratory involvement was noted in only 1 case.^13)^

Conversely, the present case of recurrent severe stridor is uncommon. A review of the literature reveals only 2 documented cases of stridor. In both cases, the esophagus was reported to be dilated. Similar to our case, the dilated esophagus may have compressed the trachea from the posterior, resulting in stridor.

An analysis of reviews of the literature and the symptoms exhibited by the present case revealed that ARSA in children also frequently results in dysphagia due to esophageal compression. In some cases, this condition arises from dysphagia, while in others, it is precipitated by a respiratory infection resulting from aspiration. In rare instances, as evidenced in the present case, it can result in recurrent severe stridor.

Currently, no established guidelines define the precise indications for surgical intervention in ARSA. Although the management of asymptomatic cases remains a topic of debate,^20)^ there is general agreement that symptomatic patients should undergo treatment. Indications for surgical intervention typically encompass dysphagia or respiratory distress, in addition to gastrointestinal bleeding associated with fistulas or aneurysms.^21,22)^ In this case, the patient experienced severe recurrent respiratory distress, making surgical intervention necessary for definitive resolution. The treatment was deemed successful, as the respiratory symptoms ultimately resolved.

## CONCLUSIONS

While ARSA is a relatively common vascular anomaly, symptomatic cases in pediatric patients are rare and can be life-threatening. Patients with feeding difficulties may subsequently develop respiratory symptoms that can be fatal. Surgical intervention should be considered for patients with feeding difficulties because anatomic structures resulting from abnormal blood vessels do not improve spontaneously.

## DECLARATIONS

### Availability of data and materials

The datasets used in this study are available from the corresponding author upon reasonable request.

## References

[1] Popieluszko P, Henry BM, Sanna B, et al. A systematic review and meta-analysis of variations in branching patterns of the adult aortic arch. J Vasc Surg 2018; 68: 298–306 e10. 28865978 10.1016/j.jvs.2017.06.097

[2] Polguj M, Chrzanowski Ł, Kasprzak JD, et al. The aberrant right subclavian artery (arteria lusoria): the morphological and clinical aspects of one of the most important variations–a systematic study of 141 reports. ScientificWorldJournal 2014; 2014: 292734. 25105156 10.1155/2014/292734 PMC4102086

[3] Derbel B, Saaidi A, Kasraoui R, et al. Aberrant right subclavian artery or arteria lusoria: a rare cause of dyspnea in children. Ann Vasc Surg 2012; 26: 419.e1–4. 10.1016/j.avsg.2011.09.007 22321486

[4] Miller JM, Miller KS. A note on the historical aspects of dysphagia lusoria. Am Surg 1992; 58: 502–3. 1642389

[5] Nedelcu AH, Lupu A, Moraru MC, et al. Morphological aspects of the aberrant right subclavian artery-a systematic review of the literature. J Pers Med 2024; 14: 335. 38672962 10.3390/jpm14040335 PMC11051064

[6] Kir M, Saylam GS, Karadas U, et al. Vascular rings: presentation, imaging strategies, treatment, and outcome. Pediatr Cardiol 2012; 33: 607–17. 22314366 10.1007/s00246-012-0187-x

[7] Medina CK, Kucera JA, Aykut B, et al. Aberrant right subclavian artery intervention can provide relief of dysphagia. Cardiol Young 2024; 34: 2583–6. 39381969 10.1017/S1047951124026532

[8] Faraj C, Essetti S, Maslouhi K, et al. Incidental finding of arteria lusoria. Glob Pediatr Health 2024; 11: 2333794X241273210. 10.1177/2333794X241273210 PMC11350531 39205859

[9] Bizhga M, Velmishi V, Sila L, et al. Dysphagia lusoria caused by aberrant right subclavian artery associated with truncus bicaroticus in an 8-month-old girl. Case report and review of literature. Pediatr Med Chir 2024; 46: 332. 10.4081/pmc.2024.332 38625064

[10] Heye T, Greiten L, Story-Hefta L, et al. Aberrant right subclavian artery: a novel approach and an overview of operative techniques. J Vasc Surg Cases Innov Tech 2023; 9: 101327. 37928561 10.1016/j.jvscit.2023.101327 PMC10624571

[11] Agarwal P, Sheridan Thompson M, Barr L, et al. Progressive dysphagia and chronic abdominal pain from vascular anomalies: a case report and literature review. JPGN Rep 2023; 4: e284. 37181925 10.1097/PG9.0000000000000284 PMC10174746

[12] Dugas J, Vozar A, Deskins SJ, et al. Infant with recurrent vomiting and poor weight gain secondary to an aberrant subclavian artery. Cureus 2023; 15: e36856. 37139276 10.7759/cureus.36856 PMC10151111

[13] Pereira AR, Grangeiro CHP, Pereira LC, et al. Oculo-auriculo-vertebral spectrum associated with aberrant subclavian artery in an infant with recurrent respiratory distress. Rev Paul Pediatr 2022; 40: e2020153. 10.1590/1984-0462/2022/40/2020153 PMC8240622 34076202

[14] Dranseika V, Erdil T, Schweiger M, et al. Dysphagia and an aberrant subclavian artery: more than just a coincidence. Interact Cardiovasc Thorac Surg 2020; 31: 228–31. 32539083 10.1093/icvts/ivaa091

[15] Fanelli U, Iannarella R, Meoli A, et al. An unusual dysphagia for solids in a 17-year-old girl due to a lusoria artery: a case report and review of the literature. Int J Environ Res Public Health 2020; 17: 3581. 32443756 10.3390/ijerph17103581 PMC7277374

[16] Nelson JS, Hurtado CG, Wearden PD. Surgery for dysphagia lusoria in children. Ann Thorac Surg 2020; 109: e131–3. 31301275 10.1016/j.athoracsur.2019.05.058

[17] Baig A, Fortner C, Rivera M, et al. Vascular anomaly: cause of infant respiratory distress and dysphagia. Respir Med Case Rep 2019; 28: 100908. 31367518 10.1016/j.rmcr.2019.100908 PMC6656703

[18] Barone C, Carucci NS, Romano C. A rare case of esophageal dysphagia in children: aberrant right subclavian artery. Case Rep Pediatr 2016; 2016: 2539374. 26904341 10.1155/2016/2539374 PMC4745392

[19] Joynt MR, Grifka RG. Spontaneous aberrant right subclavian arterio-oesophageal fistula in a previously healthy child. Cardiol Young 2015; 25: 1425–7. 25498315 10.1017/S1047951114002388

[20] Konstantinou N, Antonopoulos CN, Tzanis K, et al. Systematic review and meta-analysis of outcomes after operative treatment of aberrant subclavian artery pathologies and suggested reporting items. Eur J Vasc Endovasc Surg 2022; 63: 759–67. 35459610 10.1016/j.ejvs.2022.02.027

[21] Watanabe M, Suzuki K, Fujinaga K, et al. Postmortem diagnosis of massive gastrointestinal bleeding in a patient with aberrant right subclavian artery-esophageal fistula. Acute Med Surg 2016; 3: 139–42. 29123767 10.1002/ams2.136 PMC5667394

[22] Singha NK, Hale SJ, Kuhlman JE. Arterio-esophageal communication from a ruptured aberrant right subclavian artery aneurysm. CT diagnosis. Clin Imaging 1998; 22: 117–21. 9543589 10.1016/s0899-7071(97)00072-7

